# Anesthesia management in living-donor liver transplantation in a patient with carbamoyl phosphate synthetase deficiency: a case report

**DOI:** 10.1186/s40981-022-00558-9

**Published:** 2022-09-07

**Authors:** Hiroki Matsushita, Tetsushiro Fujiyoshi, Koichiro Yoshimaru, Toshiharu Matsuura, Yuichi Mushimoto, Yuji Karashima, Ken Yamaura

**Affiliations:** 1 Department of Anesthesiology and Critical Care Medicine, Kyushu University Hospital, 3-1-1 Maidashi Higashi-ku, Fukuoka, 812-8582 Japan; 2 Department of Pediatric Surgery, Kyushu University Hospital, 3-1-1 Maidashi Higashi-ku, Fukuoka, 812-8582 Japan; 3 Department of Pediatrics, Kyushu University Hospital, 3-1-1 Maidashi Higashi-ku, Fukuoka, 812-8582 Japan

**Keywords:** Pediatric, Urea cycle disorders, UCD, Carbamoyl phosphate synthetase deficiency, CPS1D, Hyperammonemia, Living-donor liver transplantation

## Abstract

**Background:**

Carbamoyl phosphate synthetase deficiency (CPS1D) is a urea-cycle disorder (UCD). We report successful perioperative management of pediatric living donor liver transplantation (LDLT) in a CPS1D patient.

**Case presentation:**

A 10-year-old female patient with CPS1D underwent LDLT. Proper administration of dextrose 50% and 60 kcal/kg/day with l-arginine and l-carnitine resulted in the avoidance of intraoperative hyperammonemia induced by hypercatabolism. Serum ammonia level transiently increased to 61 mmol/L in the anhepatic phase and decreased to 44 mmol/L after reperfusion.

**Conclusions:**

We suggest anesthesia management with administration of dextrose to avoid hyperammonemia during LDLT in patients with CPS1D.

## Background

Carbamoyl phosphate synthetase deficiency (CPS1D) is a congenital metabolic dysfunction caused by a urea cycle disorder (UCD). Any enzymatic defect in the urea cycle inhibits the production of essential amino acids from arginine and causes nitrogen accumulation and hyperammonemia [1]. CPS1D is diagnosed by genetic examination, and its worldwide prevalence is reportedly 1/526,000–1,300,000 live births [2]. Children with CPS1D have neurological dysfunctions, such as seizures, developmental disabilities, and behavioral abnormalities, due to uncontrolled hyperammonemia [3]. Liver transplantation (LT) is the final treatment for CPS1D patients who have difficulty in withdrawing continuous plasmapheresis for hyperammonemia [1, 4]. In contrast, the physical stress induced by LT surgery stimulates protein catabolism and cortisol production, resulting in hyperammonemia [4, 5]. Therefore, suppressing surgical stress to avoid the elevation of serum ammonia during perioperative management is important [3, 6]. We present a case of anesthesia management during living-donor LT (LDLT) in a patient with CPS1D.

## Case presentation

Informed consent for this case  report was provided by the patient’s parents.

The patient was a 10-year-old girl, 20.9 kg (−2.9 SD) by weight and 123 cm (− 3.2 SD) in height. Standard deviations of body weight and height were calculated using the growth curve presented by the National Center for Child Health and Development in Japan. In the neonatal period, she showed impaired consciousness due to hyperammonemia and underwent continuous plasmapheresis. She was diagnosed with CPS1D when she was 7 years old. Intake of sodium benzoate, arginine, and protein was restricted to prevent elevation of serum hyperammonemia. Decreasing her serum ammonia only by medicine and meal limitation was difficult, and LDLT was performed when she was 9 years old.

No remarkable abnormality or family history was found for this patient on preoperative examination. Although her liver function was preserved at Child-Pugh A, her serum ammonia level was 45 mmol/L (normal range 7.0–38.8 mcmol/L). The intake of sodium benzoate, l-arginine, l-carnitine, citrulline, lactulose, multivitamins plus zinc, dextrose, and carbamazepine 20 mg/kg/day was continued until the day of surgery. Protein intake was also restricted to 0.7–1.0 g/day. Her physical status according to the American Society of Anesthesiologists was class 4 due to uncontrolled hyperammonemia.

General anesthesia was performed with fentanyl 100 μg, propofol 50 mg, and rocuronium 20 mg for induction, and with isoflurane 2%, fentanyl 33.3 μg/kg, remifentanil 0.2 μg/kg/min, and rocuronium 5.6 mg/kg for maintenance. Arterial and central venous pressures were monitored in addition to standard physical monitors, such as electrocardiograms, percutaneous oxygen saturation, capnography, and end-tidal concentration of isoflurane. Dextrose 50% with l-arginine 200 mg/kg/day and l-carnitine 30 mg/kg/day was continuously administered, equivalent to an appropriate dose of 60 kcal/kg/day.

**Fig. 1:**
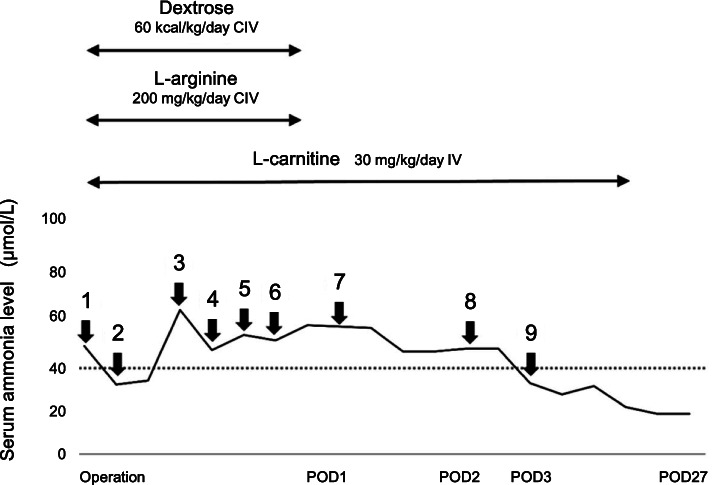
Perioperative treatment and serum ammonia levels. The serum ammonia level was 45 mcmol/L before surgery (1). Serum ammonia was 29 mmol/L after anesthesia induction (2), 61 mcmol/L in the anhepatic phase (3), 44 mcmol/L an hour after reperfusion (4), 50 mcmol/L 4 h after reperfusion (5), and 50 mcmol/L at the end of the operation (6). Serum ammonia decreased to 54 mcmol/L on POD 1 (7), 44 mcmol/L on POD 2 (8) and to the normal range on POD 3 (9). The horizontal dotted line indicates a normal level of 38.8 mcmol/L

Blood laboratory tests, including serum ammonia tests, were performed every 2 h. Serum ammonia level was 29 mcmol/L at anesthesia induction and 31 mmol/L 2 h after the start of the operation (Fig. 1). Serum ammonia transiently increased up to 61 mmol/L during the anhepatic phase, decreased to 44 mcmol/L an hour after reperfusion of the transplanted liver, and further increased to 50 mcmol/L at the end of the operation. The glucose level was 102 g/dL at anesthesia induction. When the glucose level reached 256 g/dL in the pre-anhepatic phase, 0.6 unit/h of insulin was initiated for the patient, and glucose was controlled within the normal range using insulin. The serum lactate level increased to 32 mg/dL in the anhepatic phase and it decreased to 22 mg/dL after reperfusion.

The operation time was 625 min, and the anesthesia time was 717 min without any complications. The total output was 865 g of blood loss and 1100 mL of urine. The total intake was 2878 mL of crystalloids and 280 mL of blood cells. Freshly frozen plasma and platelets were not used in this study. Postoperatively, the patient was transferred to the ICU under midazolam and fentanyl anesthesia. The infusion of l-carnitine and dextrose was continued subsequently (Fig. 1). Serum ammonia levels decreased to 54 mcmol/L on POD 1 and 44 mcmol/L on POD 2, and they finally decreased to the normal range on POD 3. The postoperative condition was good, and the patient was transferred to the general ward on POD 3 and discharged on POD 27.

## Discussion

Here, we report a case of LDLT in a patient with CPS1D, which was a congenital metabolic dysfunction of the urea cycle. Any enzymatic defects in the urea cycle inhibit the production of essential amino acids from arginine and cause nitrogen accumulation and hyperammonemia in patients with CPS1D. According to the guidelines for UCD treatment, dextrose and arginine should be administered to suppress protein catabolism and avoid hyperammonemia, in addition to low-protein meals [1, 3, 4]. Although LT is indicated as a final treatment in patients with severe UCD, hyperammonemia is caused by surgical stress [5]. Therefore, perioperative anesthesia management is suggested to suppress the increase in serum ammonia levels during the perioperative period.

Serum ammonia levels should be checked frequently to avoid hyperammonemia during LDLT. Some reports have shown that serum ammonia level is an essential parameter for evaluating transplanted liver function [7]. In adult LDLT for end-stage liver dysfunction, serum ammonia increases transiently during the anhepatic phase and returns to normal levels after reperfusion when the transplanted liver starts reworking. The normalization of serum ammonia indicates a well-functioning liver graft in the acute phase of LT, and [8] increased serum ammonia over 200 mcmol/L causes cerebral edema and intracranial hypertension; therefore [1], hyperammonemia should be avoided in such patients. In our case, serum ammonia was measured every 2 h, and critical hyperammonemia was not observed during surgery. Some reports have suggested that serum ammonia levels should be used as a standard monitor for LT [3, 9, 10].

No clinical guidelines exist for anesthetic management in pediatric LT with UCD, and our perioperative anesthesia management plan was based on the guidelines for UCD and hyperammonemia, such as the Middle East Hyperammonemia and Urea Cycle Disorders Scientific Group and the Japanese Society for Inherited Metabolic Disease [1, 4, 11]. Guidelines indicate that the proper administration of dextrose and l-arginine is effective in preventing hypercatabolism. If hyperammonemia occurs, calorie intake should be increased up to 110% of the standard daily intake with dextrose to reduce the endogenous protein consumption and l-arginine production [11]. Following the guidelines, we planned to increase dextrose intake to 100 kcal/kg/day with l-arginine in cases where serum ammonia was critically increased in the perioperative period. Continuous renal replacement therapy (CRRT) has also been used to treat severe hyperammonemia, although its effectiveness during surgery has not been reported till date. Intraoperative CRRT induction should be suggested if serum ammonia exceeds 500 mcmol/L and any medical treatment is ineffective in controlling serum ammonia [12]. Fortunately, hyperammonemia did not occur and additional dextrose intake or CRRT was not performed in our case.

According to the guidelines, dextrose with l-arginine was continuously administered during the operation to avoid hyperammonemia. However, it is unclear how to decrease and complete dextrose administration. The transplanted liver begins to function, and the urea cycle is functional following reperfusion. In addition to serum ammonia, platelets, and lactate are useful markers of transplanted liver function [13]. In this case, the normalization of serum ammonia, platelets, and lactate might have indicated the possibility of decreasing dextrose administration. Dextrose and l-arginine administration were continued during surgery, even if serum ammonia started to decrease after reperfusion of the transplanted liver. When serum ammonia, platelet, and lactate levels were maintained at normal levels in the ICU, we decided to complete dextrose administration. Serum ammonia levels were within the normal range without dextrose after LT. Although no reports have shown an optimal program to decrease dextrose administration after LT, serum ammonia, platelets, and lactate levels might be relevant markers to indicate a decrease in dextrose after LT in CPS1D patients.

Some reports have shown that a smaller amount of blood loss and blood infusion contributes to a good outcome in LT [14]. Blood products contain serum ammonia, and blood transfusions increase the serum ammonia levels [15, 16]. Furthermore, blood transfusions are associated with increased graft failure and mortality rates [17, 18]. Compared to patients with biliary atresia, liver function and coagulation activity were generally maintained in children with CPS1D. In our case, liver function and coagulation activity were preserved, and the blood infusion required was only 13 mL/kg of red blood cells. Therefore, a low-dose blood infusion may contribute to a good outcome in LDLT.

In conclusion, we suggest anesthesia management with administration of dextrose to avoid hyperammonemia during LDLT in patients with CPS1D.

## Data Availability

Not applicable

## Abbreviations

ICU: Intensive care unit
POD: Post-operative day
CRRT: Continuous renal replacement therapy
CIV: Continuous intravenous injections
IV: Intravenous injection
